# Dr. Playfair on Mediævalism at the Royal Colleges

**Published:** 1898-03-26

**Authors:** 


### DR. PLAYFAIR ON MEDIÆVALISM AT THE ROYAL COLLEGES.

In his Valedictory Lecture delivered at King's College, on resigning the chair of Obstetric Medicine, Dr. Playfair touched upon many things, but upon few more happily than on the Royal Colleges, their Fellows, and their diplomas.

Everyone, he says, who has the honour to belong to the College of Physicians is proud of it, and fond of it, but it is a very ancient institution, and like other ancient institutions, it is not free from traditions and prejudices that are not altogether wise. Moreover, it has a good many Fellows who are by nature fine old crusted Tories, whom everyone loves and respects, who would like to keep the college very much as it was in the time of Henry VIII. Possibly some of these may still object to obstetric physicians operating when they have an aptitude for doing so, and have such work to do. But if such views are held, as is not unnatural, they have never done any practical harm. . . . Of the views on this subject of one of the wisest and most liberal-minded of its presidents, the late Sir Andrew Clark, there can be no doubt, for I have often heard him say, that a man should do exactly what he thought himself most capable of doing, and I have had practical proof of it by having on several occasions operated on gynæcological cases he sent to me for the purpose.

But I am told that there is a cloud, at present no bigger than a man's hand, rising in another quarter. I am given to understand that our friends the hospital surgeons—or some of them—dismayed, let us hope, by the operative successes of their obstetrical colleagues, are beginning to clamour for protection, and are agitating for the enactment of a rule that no obstetrician is to operate unless he is a Fellow of the Royal College of Surgeons. But a man's skill as an operator depends on his brains and on his fingers, not on the letters which he writes behind his name. "I know Fellows of the College of Surgeons whom I would not trust to vaccinate a baby, and I know Fellows of the College of Physicians to whom I would unhesitatingly confide any operation they chose to undertake." We boast, he says, of belonging to a country which has been wise enough to adopt free trade in commerce, yet we talk of adopting a protective measure which could not find a parallel in any other country of the world, in none of which do such mediæval distinctions between different branches of the profession exist. The plain fact is, and it is one which cannot be got over, that the evolution of medicine has produced a class of practitioners who are and must be "physicians and surgeons" in a higher sense, and the sooner sensible and unprejudiced men reconcile themselves to their existence the better.

We cannot but feel that there is a ring of common sense in all this which should attract the attention of those who possess influence and power to sway the councils of our profession. It is a very serious matter that small and, perhaps, trivial circumstances in the earlier part of a man's career as a student, little errors of judgment in the arrangement of his curriculum, should influence his whole life, and should practically debar him from appointments for which he may later on develop special aptitudes—aptitudes undreamed of at the time when he chose, or, perhaps, when his father, or his guardian, or his schoolmaster chose for him, the diploma for which he should work.

